# Chronic Disease Epidemiology and Control, 3rd Edition

**Published:** 2010-12-15

**Authors:** Keila Y. Torres

**Affiliations:** Drexel University, Philadelphia, Pennsylvania

**Figure:**
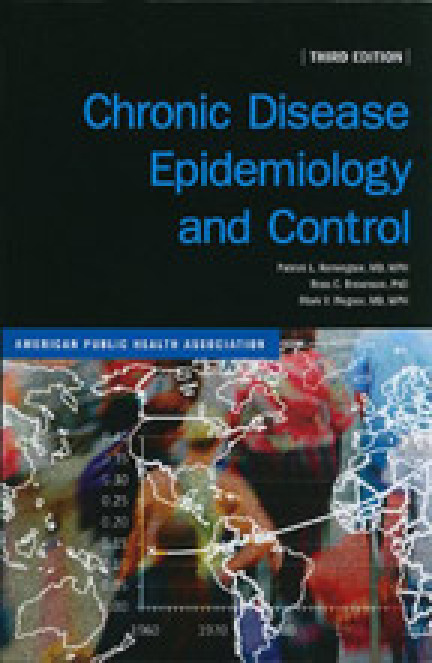


The third edition of *Chronic Disease Epidemiology and Control* presents an updated compendium of contributions from a diverse group of public health professionals with expertise in chronic disease causation, prevention, and intervention. The book targets varied readers, from those in academia to those in public health practice. It provides a well-organized overview of the life course of major chronic diseases. Matthew McKenna and Janet Collins set the foundation for the importance of the topic by stressing that “the course of a chronic disease can be viewed as a continuum from the ‘upstream’ social and environmental determinants, to behavioral risk factors, chronic conditions, chronic diseases, and, finally, impairment, disability and ultimately death.”

The overview discusses social determinants leading to risk factors for chronic conditions. The book is divided into 4 sections that help guide the reader through the chronic disease continuum: public health approaches, selected chronic disease risk factors, major chronic conditions, and major chronic diseases. Each section offers an objective and neutral discussion of featured topics. Public health approaches, for example, deal with issues and challenges in chronic disease control, epidemiologic methods, interventions, and surveillance. The authors comprehensively cover current knowledge, evidence-based best practices, and suggestions for future research related to the evolution of major chronic diseases.

The book’s intended audience is most likely familiar with the subject matter. Some chronic disease experts may feel that this is all familiar terrain because the discussion centers on the more widespread risk factors and conditions. However, the strength of the book is its organization and layout. The editors and contributing writers have provided an efficiently organized and comprehensive overview of the most salient chronic disease issues, making for easy reading. Each section stands alone but flows to the next coherently; the reader is barely aware that the chapters of each section are written by different people.

The intrinsic value of this thoughtful and up-to-date collection is its usefulness as a reference for readers who are navigating the nuances of chronic diseases issues. As a member of the target audience, I gained a better understanding of the life course trajectory of major chronic diseases and was reminded of the well-known but often forgotten notion that chronic diseases are preventable rather than inevitable.

